# Expression optimization, purification and *in vitro* characterization of human epidermal growth factor produced in *Nicotiana benthamiana*

**DOI:** 10.1016/j.btre.2020.e00524

**Published:** 2020-09-05

**Authors:** Oranicha Hanittinan, Yamin Oo, Chatchai Chaotham, Kaewta Rattanapisit, Balamurugan Shanmugaraj, Waranyoo Phoolcharoen

**Affiliations:** a Department of Pharmacognosy and Pharmaceutical Botany, Faculty of Pharmaceutical Sciences, Chulalongkorn University, Bangkok, Thailand; b Research Unit for Plant-produced Pharmaceuticals, Chulalongkorn University, Bangkok, Thailand; c Department of Biochemistry and Microbiology, Faculty of Pharmaceutical Sciences, Chulalongkorn University, Bangkok, Thailand; d Cell-based Drug and Health Products Development Research Unit, Faculty of Pharmaceutical Sciences, Chulalongkorn University, Bangkok, Thailand

**Keywords:** Human epidermal growth factor, *Nicotiana benthamiana*, Plant-produced protein, Recombinant protein, Transient expression

## Abstract

Human epidermal growth factor (hEGF) has gained clinical importance due to its ability to promote wound healing. Due to its commercial applications and high market demand, recombinant EGF has been produced in several forms. Currently, plant expression system is considered as potential alternative for low-cost recombinant protein production. Hence, this study focused on improving the production of hEGF in plants by effective gene construct design and optimizing the *Agrobacterium* culture conditions for high protein production. In this context, hEGF gene was cloned into plant geminiviral expression vector pBYR2e and transformed in to *N. benthamiana* leaves *via*., agroinfiltration. The recombinant hEGF was purified from the plant crude extracts by single-step affinity chromatography. Furthermore, the plant-produced hEGF has shown to promote cell migration comparable to commercial hEGF in HaCaT cells *in vitro*. These results indicated the potential of plant expression system for the production of recombinant hEGF for tissue engineering applications.

## Highlights

• Growth factors play a crucial role in tissue repair and wound healing.
• Recombinant human epidermal growth factor was produced in *N. benthamiana* by agroinfiltration.
• Expression conditions were optimized and the recombinant protein was purified.
• Plant-produced hEGF facilitate the HaCaT cell proliferation and cell migration *in vitro.*
• Plant-produced hEGF can potentially be exploited for their application in tissue engineering.

## 1 Introduction

Epidermal growth factor (EGF) is a single-chain polypeptide with 53 amino acids, that has been known to promote the growth of epidermal cells and keratinocytes [1]. EGF was first identified in the submaxillary salivary glands of adult mice [2] and also detected in skin, tears, milk, saliva, urine, plasma and other body fluids [3]. It has distinct roles in healing of injuries and skin rejuvenation including burns, alkali-burned corneas, chronic diabetic foot ulcers [4,5]. Further, EGF has also shown to be effective and safe for the treatment of radiation-induced mucositis [6].

The market demand of hEGF is high due to its several biological effects and applications, hence recombinant hEGF had been produced in a wide range of cell-based platforms including *Escherichia coli* [7], *Bacillus brevis* [8], yeast *Saccharomyces cerevisiae* [9], *Pichia pastoris* [10], and human embryonic kidney (HEK) cells [11], *etc.* Although prokaryotic expression is the most employed and preferred production system for recombinant protein production, the lack of post-translational machinery in bacterial cells is one of the major limitations, as it might adversely affect protein stability and its biological activity [12].

Recently, plants are utilized as an alternative protein production platform due to its advantages in terms of low production cost [13], high scalability [14], rapid production speed, low contamination risk of human pathogens and the ability to perform post-translational modifications which is essential for the structural and functional integrity of the proteins [[15], [16], [17]]. Moreover, *Nicotiana*-based system is a member of leaf-based systems which has desirable attributes, *i.e.,* readily modifiable to genetic engineering approaches, well-established transformation protocol, non-food crop, all-year season harvesting and growth [15,18]. *N. benthamiana* is a preferred host for the production of functional recombinant proteins such as cytokines, enzymes, therapeutic proteins, antibodies, and vaccines [15,19]. However, some of the limitations of plant-based platform includes low expression levels and difficulties in downstream processing/purification which have an immense effect on both cost and quality of the plant-produced proteins [20]. These limitations need to be addressed in plant expression platform in order to compete with the conventional expression system. Earlier studies have attempted different strategies to maximize the hEGF production in plants. Wirth et al. 2004 [21] expressed hEGF gene constructs targeting to different sub-cellular organelles. The apoplast targeting of hEGF showed the protein yields of up to 0.11 % total soluble protein (TSP) whereas the protein was barely detectable when expressed in cytoplasm. Bai et al. (2007) suggested that the codon optimization of foreign genes and addition of C-terminal ER retention signal peptide (KDEL) during gene and construct design could improve the expression of heterologous protein in plants. With optimized codons and ER targeting signal, hEGF expression was obtained upto 0.3 % total soluble protein (TSP) in transgenic tobacco [22]. Thomas et al.(2014) achieved the hEGF yield of up to 6.24 % of TSP in *Nicotiana benthamiana* by optimizing different parameters such as sub-cellular targeting, codon optimization and the use of a silencing inhibitor [23].

The protein extraction is the first step in the recombinant protein purification which is based on disruption of harvested plant leaves by blending and further clarification to collect the clear crude extract. However, ribulose-1,5-bisphosphate carboxylase/oxygenase (known as RuBisCO), which is the most abundant plant protein, other host contaminants, plant tissues along with protein of interest have been released into crude extract which could interfere with the purification efficiency [24]. Hence, in this study, different approaches were employed for optimization of hEGF expression in *N. benthamiana*. The effect of recombinant hEGF expression with regard to gene construction, position of histidine (His) tag (N- or C-terminus), and sub-cellular targeting were examined. The transformation efficiency was improved by optimizing *Agrobacterium* cell concentration and an attempt was made to improve the recombinant protein quality. The biological activity of the plant-produced hEGF was tested on human keratinocyte (HaCaT cells) for its cell proliferation and cell migration effect.

## 2 Materials and methods

### 2.1 Cloning of hEGF gene into plant expression vector

**Fig. 1:**
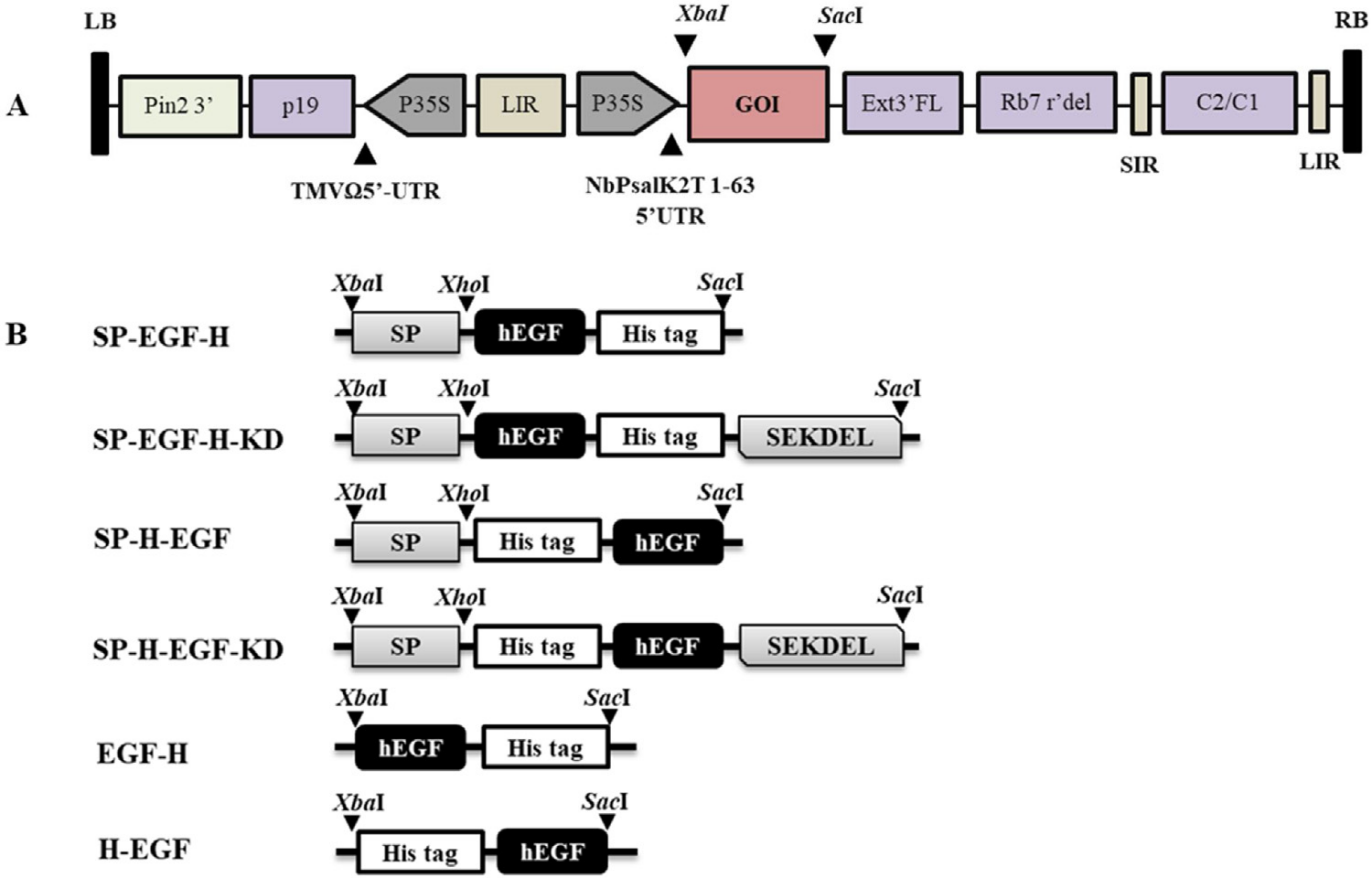
Schematic representation of different plant expression vectors used in the present study: (A) Geminiviral vector was used for transient expression of hEGF in *Nicotiana benthamiana*. P35S: Cauliflower Mosaic Virus (CaMV) 35S promoter, TMVΩ 5′-UTR: 5′ untranslated region of tobacco mosaic virus Ω, hEGF gene: hEGF coding sequence, Ext3′ FL: 3′ full length of tobacco tabacum extention gene, SIR: short intergenic region of BeYDV genome, LIR: long intergenic region of BeYDV genome, C2/C1: Bean Yellow Dwarf virus (BeYDV) ORFs C1 and C2 which encode for replication initiation protein (Rep) and RepA, PNOS: nopaline synthase promoter, P19: P19 gene from Tomato Bushy Stunt Virus (TBSV), Nos3′: 3′ termini of the polyadenylated nos mRNA. (B) Six different hEGF gene constructs used in this study. SP: signal peptide at N-terminal, His tag: His tag residues at either N-terminus or C-terminus, hEGF: human epidermal growth factor, SEKDEL: C-terminal endoplasmic reticulum (ER) retention signal peptide. Arrowhead indicates the sites of restriction enzyme used for gene cloning (For interpretation of the references to colour in this figure legend, the reader is referred to the web version of this article).

The nucleotide sequence encoding for human epidermal growth factor (hEGF) (Genbank accession no. :AFA26280.1) was codon-optimized for *N. benthamiana* by GeneArt gene synthesis (Thermoscientific). In order to optimize the recombinant gene expression in plants, the hEGF gene was generated into six different gene constructs as shown in Fig. 1. Briefly, the construct designated as “SP-EGF-H” and “SP-EGF-H-KD” flanked with an N-terminal signal peptide (SP) and a C-terminal 8xHistag (H) with or without SEKDEL (KD). The “SP-H-EGF” and “SP-H-EGF-KD” construct was fused with a signal peptide, His tag at N-terminal and either with or without SEKDEL at C-terminal. Moreover, the hEGF gene was also generated without N-terminal signal peptide and His tag on either C or N-terminal, designated as “EGF-H” and “H-EGF” respectively. The synthetic construct was digested with *Xba*I and *Sac*I restriction enzymes (BioLabs, USA), gel extracted and ligated into a geminiviral expression vector (pBYR2eK2Md) by using T4 DNA ligase (Biolabs, USA). The ligated plasmid was transformed to *E.coli* strain DH10B *via.,* heat shock method and selected on the Luria-Bertani (LB) agar plates supplemented with appropriate antibiotics. The recombinant clones were confirmed by polymerase chain reaction (PCR) using gene specific primers. The PCR cycling conditions were as follows: initial denaturation at 98 °C for 2 min followed by 30 cycles of 98 °C for 30 s, 52 °Cfor 30 s, and 72 °C 30 s, with final elongation step at 72 °Cfor 10 min.

### 2.2 Determination of optimal gene construct

Expression vectors harboring different hEGF constructs were mobilized into *A. tumefaciens* strain GV3101 *via*., electroporation. *Agrobacterium* clones were confirmed by PCR analysis as described above. Recombinant *A. tumefaciens* was inoculated in LB media supplemented with 50 μg/mL each of kanamycin, gentamicin, rifampicin and incubated at 28 °C. The overnight grown *Agrobacterium* cells were pelleted by centrifugation (6000 rpm for 10 min) and the pellet was resuspended with infiltration buffer (10 mM 2-N-morpholino-ethanesulfonic acid (MES) and 10 mM MgSO_4_, pH5.5) to get the final OD_600_ at 0.4. The wild-type *N. benthamiana* plants grown under controlled conditions with 8 h dark/16 h light cycle at 28 °C for 6–8 weeks was used for agroinfiltration. Recombinant *A. tumefaciens* were infiltrated into *N. benthamiana* plants by using a syringe without needle (Syringe infiltration).

In order to compare the expression levels of different constructs, leaves were infiltrated with *Agrobacterium* harboring either one of the constructs. The infiltrated leaves were harvested and extracted with extraction buffer (5 mM Imidazole, 20 mM Tris−HCl, 50 mM NaCl, pH 7.4). Clear crude extracts were obtained after centrifugation at 13,000 rpm for 30 min. Each protein sample was analyzed by Sodium dodecyl sulfate-polyacrylamide gel electrophoresis (SDS-PAGE) and Western blot. Briefly, clarified crude extract was mixed with reducing loading buffer (125 mM Tris−HCl pH 6.8, 12 % SDS, 10 % glycerol, 22 % β-mercaptoethanol, and 0.001 % bromophenol blue, pH 6.8) and denatured at 95 °C for 5 min. The protein samples were separated on 6–18 % polyacrylamide gels and stained with coomassie blue stain (AppliChem, Germany). For Western blot analysis, the protein was transferred to PVDF membrane (Immun-Blot® PVDF, Bio-Rad) at 75 V for 90 min. Then, the membranes were blocked with 5% non-fat skim milk in PBS pH 7.4 for 1 h and then incubated with rabbit polyclonal anti-His antibody conjugated to horseradish peroxidase (HRP) (Abcam, UK) at 1:5,000 dilution in 3% non-fat skim milk for 2 h or overnight at 4 °C. The blots were developed by chemiluminescence using ECL plus detection reagent (GE Healthcare, UK).

### 2.3 Optimization of *Agrobacterium* transformation in *N. benthamiana*

Recombinant *A. tumefaciens* containing pBYR2e-hEGF were pelleted and resuspended in infiltration buffer to get a final OD_600_ of 0.2, 0.4, 0.6 and 0.8. The different concentrations of cells were infiltrated in the same leaves of *N. benthamiana* plants. At least three plants were tested with each condition to mitigate the variation of leaf location and batch-to-batch variation of plants. Then, the leaves were collected on 4 day post-infiltration (dpi) and the plant crude extracts were collected and analyzed by Western blot.

### 2.4 Purification of plant-produced hEGF

For large scale protein production, vacuum infiltration was performed by placing the plants upside down and submerging the leaves into the infiltration buffer containing *Agrobacterium*-harboring the hEGF gene construct. Infiltrated leaves were harvested and grinded with extraction buffer. The supernatant was collected by centrifugation at 13,000 rpm for 30 min and clarified by filtration by using a sterile 0.45-micron filter before loading into Nickel-nitrilotriacetic (Ni-NTA) affinity resin (Amintra®). Briefly, the column was washed with 10 column volumes of wash buffer (5 mM and 20 mM Imidazole, 20 mM Tris−HCl, 50 mM NaCl, pH 7.4, respectively) and eluted the recombinant protein with elution buffer (250 mM Imidazole, 20 mM Tris−HCl, 50 mM NaCl, pH 7.4). The purified protein samples were analyzed by SDS-PAGE, Western blot, and quantified by ELISA. The total soluble protein (TSP) in the plant crude extracts was estimated by using Bradford assay (Bio-Rad) by following manufacturer’s instruction.

### 2.5 Preliminary study on optimization of downstream processing for hEGF purification

#### 2.5.1 Effect of extraction volume

In order to assess the effect of extraction volume on protein purification, twenty gram of leaves were grinded with liquid nitrogen in a mortar and pestle. The leaf powder was extracted with 2 mL of extraction buffer per gram of leaf fresh weight. After extraction, the extract was divided into two sets. The first set (1:2) was centrifuged at 13,000 rpm at 4 °C for 30 min; whereas the extract in the second set (1:10) was further diluted and vortexing for 5 min before centrifugation. Both the clarified extracts were loaded into the gravity flow column containing Ni-NTA resin and purified by affinity column chromatography. The purified protein was analyzed by SDS-PAGE and Western blot analysis.

#### 2.5.2 Effect of ammonium sulfate precipitation

To improve the protein purification efficiency, the clarified crude extract was added with varying concentrations of ammonium sulfate (30–80 %) to remove host cell proteins. Briefly, 30 % ammonium sulfate was added to the clarified plant extract and incubated for 30 min at 4 °C with constant stirring. After centrifugation at 13,000 rpm for 30 min at 4 °C, the supernatant was collected and ammonium sulfate was added up to 40, 50, 60, 70, or 80 % repeatedly. The pellet was resuspended with extraction buffer and analyzed by SDS-PAGE and Western blotting.

#### 2.5.3 Effect of extraction buffer pH

One gram of harvested leaves was grinded with extraction buffer with varying pH ranging from 4 to 8. Then, the extract was centrifuged at 13,000 rpm for 30 min and the supernatant was collected. The clarified protein was analyzed by SDS-PAGE and visualized by coomassie blue staining. For Western blot analysis, the protein was transferred to PVDF membrane and detected by using anti-His antibody.

### 2.6 Protein quantification by ELISA

The concentration of plant-produced hEGF was quantified by using sandwich ELISA (Human EGF Duo Set ELISA kit, R&D system, USA). Briefly, 96 well plates were coated with 50 μL of mouse anti-human EGF capture antibody (in PBS) and incubate at 4 °C overnight. Plates were washed with PBST (0.05 % tween-20 in PBS, pH 7.2–7.4) and blocked with 200 μL 1% BSA in PBS for 1 h at room temperature (RT). Following the washing step, 50 μL of the plant-produced hEGF protein and standard (diluted in 1% BSA in PBS) was added into each well and incubated for 2 h at RT. After washing, 50 μL of biotinylated goat anti-human EGF detection antibody was added and incubated for 2 h at RT. Then the plates were washed thrice and 50 μL of streptavidin-HRP B was added in each well and incubated for 20 min in dark at RT followed by washing with PBST thrice. TMB substrate was added and left to develop the color before terminating the reaction using 50 μL of 2 N H_2_SO_4_. Plates were measured the optical density at 450 nm using a microplate reader (SpectraMax® M5, USA).

### 2.7 Chemical reagents

Methyl thiazolyl diphenyl-tetrazoliumbromide (MTT), Hoechst 33,342, propidium iodide (PI), dimethylsulfoxide (DMSO) was purchased from Sigma-Aldrich Chemical (St. Louis, USA). Ammonium sulfate ((NH_4_)_2_SO_4_), potassium dihydrogen phosphate (KH_2_PO_4_) are derived from Merck (Darmstadt, Germany). Di-sodium hydrogen orthophosphate anhydrous (Na_2_HPO_4_), potassium chloride (KCl) and sodium chloride (NaCl) are obtained from Univar (Ajax, Australia). Trypsin-EDTA (0.25 %) was purchased from Gibco (Gaithersburg, USA) and commercial recombinant human EGF was purchased from R&D system (USA).

### 2.8 Cell line and culture

Human keratinocyte HaCaT cells obtained from Cell Lines Service (CLS, Heidelberg, Germany) were cultured in Dulbecco’s Modified Eagle’s Medium (DMEM) supplemented with 10 % fetal bovine serum (FBS), 2 mmol/L of l-glutamine and 100 units/mL of penicillin/streptomycin solution (Gibco, Gaithersburg, MA, USA). Cells were incubated at 37 °C in a humidified incubator with 5% CO_2_ until reaching 70–80 % confluency before passaging for further experiments.

### 2.9 Cytotoxicity assay

Cytotoxicity assay was performed by MTT assay to evaluate cell metabolic levels after treatment with plant-produced hEGF. Human keratinocyte cells were seeded in a 96-well plate at the density of 1 × 10^4^ cells per well and incubated for 24 h. After incubation, cells were treated with various concentrations (5, 10, 50, and 100 ng/mL) of plant-produced hEGF and commercial hEGF for 24 h. Then, the cells were incubated with 0.4 mg/mL MTT reagent for 3−4 h at 37 °C. After incubation, the MTT reagent was replaced with 100 μL DMSO to dissolve the formazan crystals. The purple formazan color was determined by a microplate reader (Perkin Elmer Microplate reader #1) at the absorbance of 570 nm.

### 2.10 Detection of mode of cell death

Co-staining with Hoechst 33342 and PI was used to determine the apoptosis and necrosis of cell death. HaCaT cells were cultured in 96-well plate at the density of 1 × 10^4^ cells per well and incubated overnight. Then the cells were treated with 50, 100 ng/mLof plant-produced hEGF and commercial hEGF for 24 h. The treated and untreated cells were incubated with the Hoechst 33342 (10 μM) and PI (5 μg/mL) for 30 min at 37 °C. The mode of cell death was visualized by fluorescent microscope (Olympus IX51 Invert Microscope #2). Hoechst 33342 stains the condensed chromatin in apoptotic cells which can be characterized by blue fluorescence and PI can only be permeable into dead cells which can be detected by red fluorescence [25].

### 2.11 Cell proliferation assay

Proliferation of viable cells was evaluated by staining with crystal violet to stain nuclei of adherent cells although staining can bind both viable and dead cells. Briefly, HaCaT cells were seeded at the density of 2 × 10^3^ cells per well in a 96-well plate. Then, the cells were incubated with 50, 100 ng/mL of hEGF for 24, 48, and 72 h. After each time point, the cells were washed to remove detached cells. The remaining viable cells were fixed with 1% (w/v) formaldehyde for 30 min. After that, the cells were stained with 0.05 % w/v crystal violet solution for 30 min at RT and then washed and air-dried. The crystal violet stained cell was solubilized with methanol (200 μL/well) for 15 min. The color intensity was measured at 570 nm using a microplate reader (Perkin Elmer Microplate reader #1).

### 2.12 Cell migration assay

Cell migration assay was performed by using *in vitro* scratch assay. HaCaT cells were seeded into 96-well plate at a density of 3 × 10^4^ cells/well and incubated overnight at 37 °C. The wound area was scratched with a pipette tip. Then, the cells were treated with 50 and 100 ng/mL of plant-produced and commercial hEGF. The width of the wound area was monitored at 0, 12, and 24 h incubation by using Nikon Ts2 inverted microscope (Magnification, 10x). Relative migration was calculated by the average distance changes in wound area of hEGF treated cells divided by the non-treated control group at 0 h time point.

### 2.13 Statistical analysis

All values are presented as mean ± SD from three independent experiments. Statistical analysis was performed using one-way analysis of variance (ANOVA), followed by Tukey’s post-hoc analysis with a P value less than 0.05 (p ≤ 0.05) considered as statistical significance.

## 3 Results

### 3.1 Evaluation of different gene constructs for effective protein production

**Fig. 2:**
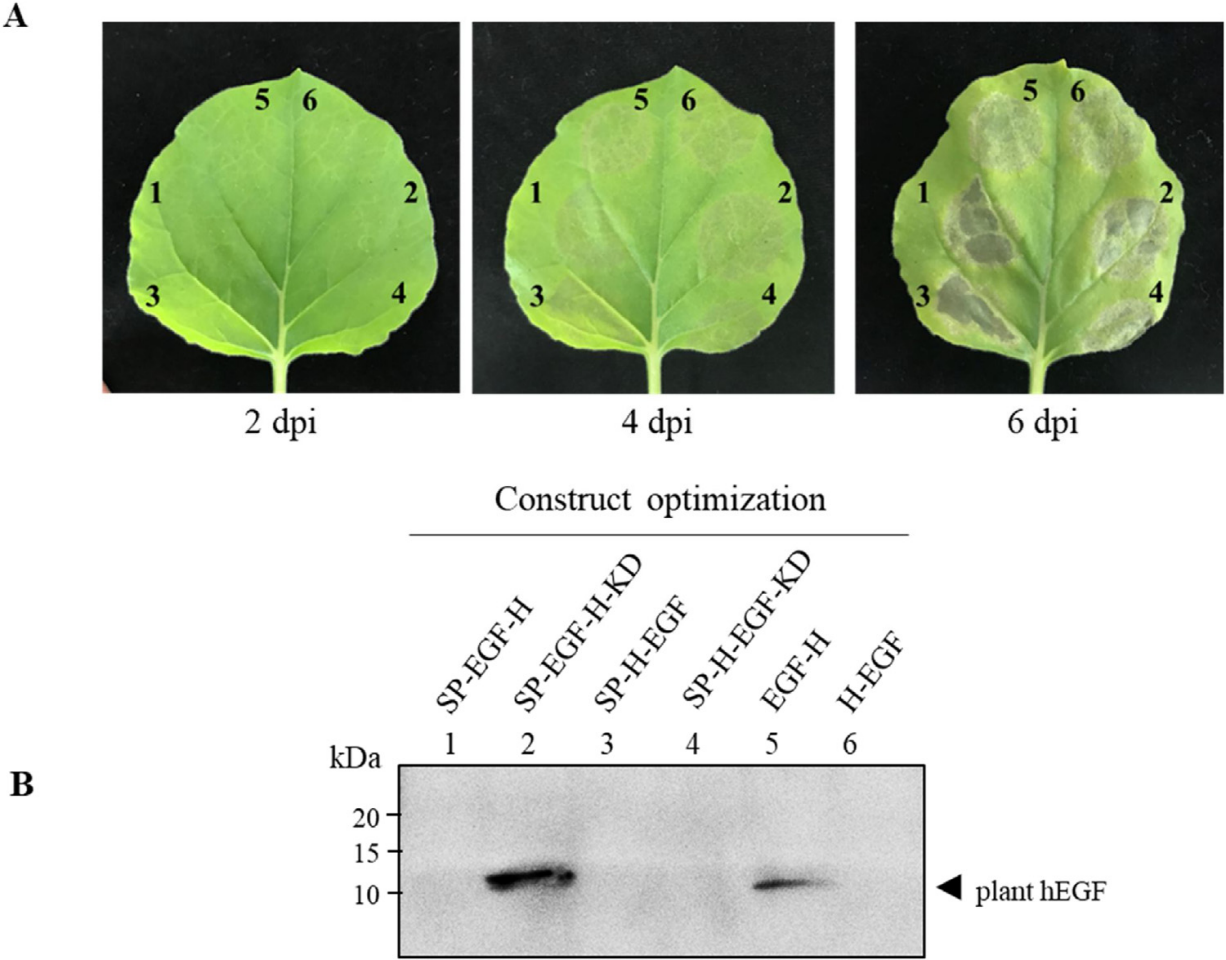
Effect of different gene constructs on recombinant hEGF production in plants (A) Typical phenotype of *N.benthamiana* leaves showing necrosis symptoms after infiltration with six different constructs on 2, 4 and 6 dpi. The leaves were infiltrated with *A. tumefaciens* strain GV3101 containing either one of the six gene constructs. (B) Western blot of plant-produced hEGF crude extract derived from leaves infiltrated with 6 different constructs under reducing condition. The protein was transferred to PVDF membrane and the blot was probed with HRP-conjugated anti-His antibody. kDa: kilodalton; Lane 1: SP-EGF-H; Lane 2: SP-EGF-H-KD; Lane 3: SP-H-EGF; Lane 4: SP-H-EGF-KD; Lane 5: EGF-H; Lane 6: H-EGF. Arrowhead represents plant-produced hEGF. Equal amounts of total soluble protein were loaded in each lane.

The nucleotide sequence of human epidermal growth factor (hEGF) was cloned into plant expression vector and six different constructs were developed as shown in Fig. 1A and B. In order to determine the optimal gene construct for hEGF expression, leaves were infiltrated with *A. tumefaciens* harboring either one of the six different constructs. After agroinfiltration, leaf necrosis was observed on day 4, in particular the leaves infiltrated with the constructs fused with N-terminal signal peptide showed high necrosis after day 6 (Fig. 2A). The infiltrated leaves were harvested and homogenized with extraction buffer on 4 dpi. The crude extract collected after infiltration with each construct was analyzed by Western blot probed with anti-His antibody (Fig. 2B). Among the six constructs tested, the results showed that the protein yield was higher in the leaves infiltrated with the construct “SP-EGF-H-KD” compared to other constructs. Hence, we have used this construct for further experiments.

### 3.2 Optimization of transformation efficiency for transient expression of hEGF in *N. benthamiana*

**Fig. 3:**
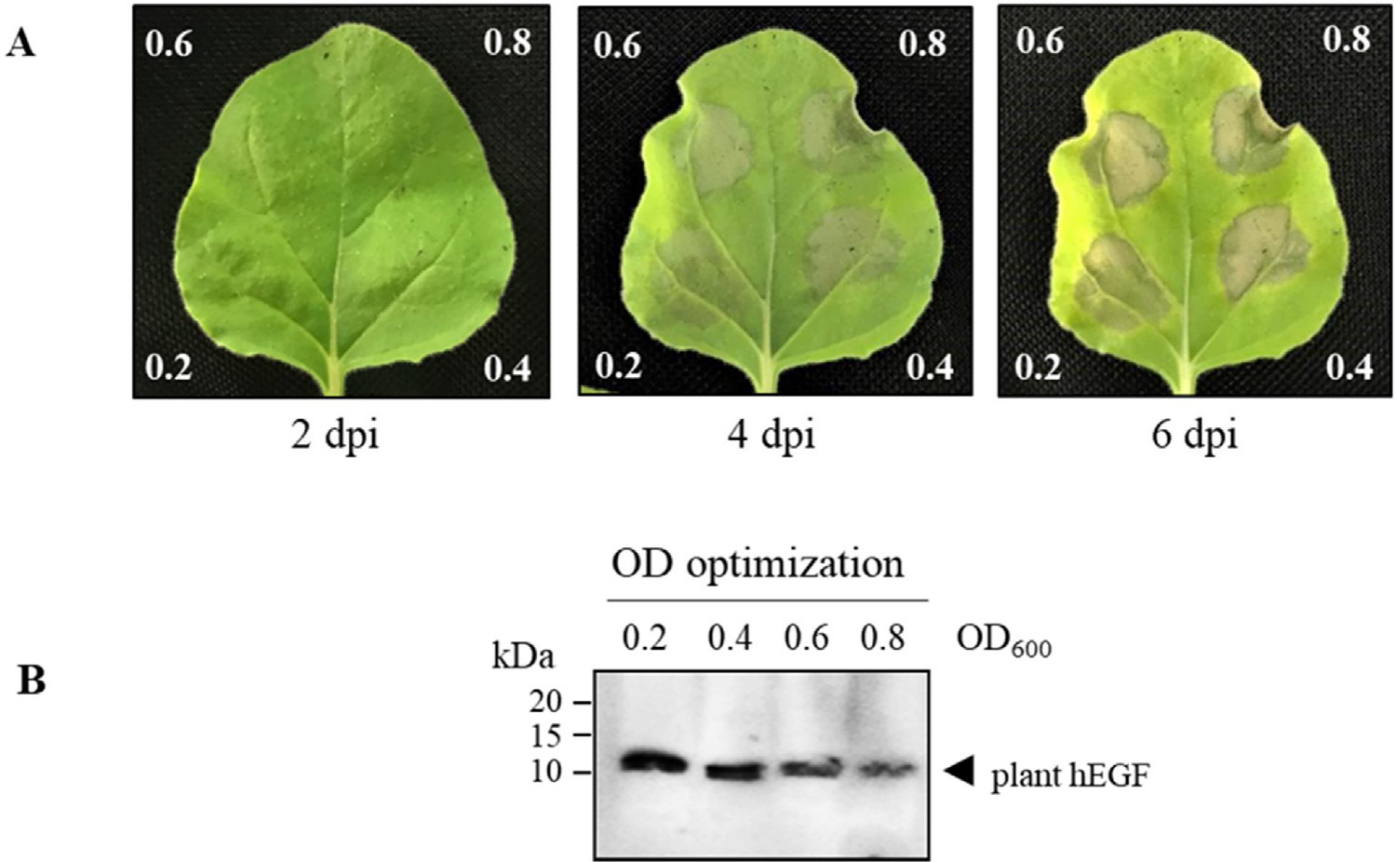
Optimization of *A. tumefaciens* concentration (A) Typical phenotype of *N.benthamiana* leaves expressing hEGF infiltrated with different *Agrobacterium* concentration (0.2, 0.4, 0.6, and 0.8 at OD_600_) on 2, 4 and 6 dpi. (B) Western blot of harvested leaves (4 dpi) infiltrated with varying concentrations of *A. tumefaciens* under reducing condition. The membrane was probed with HRP-conjugated anti-His antibody. kDa: kilodalton; OD_600_:optical density at 600 nm; dpi: day post-infiltration.; the lane number represents the OD_600_ of *Agrobacterium cells* used for agroinfiltration. Arrowhead represents plant-produced hEGF. Equal amounts of total soluble protein were loaded in each lane.

Transformation efficiency was optimized to further increase the hEGF expression in plants. The influence of *Agrobacterium* cell density on transformation efficiency was investigated by agroinfiltration with varying *Agrobacterium* cell density (0.2, 0.4, 0.6, and 0.8) at OD_600_. As shown in Fig. 3, efficient transformation was observed when low concentration of *Agrobacterium* (0.2) was used; whereas high cell density (0.8) showed the lowest transformation efficiency. Thus, the hEGF transformation into *N. benthamiana* requires moderate *Agrobacterium* concentration for optimal gene expression.

### 3.3 Purification of hEGF from *N. benthamiana* leaves

**Fig. 4:**
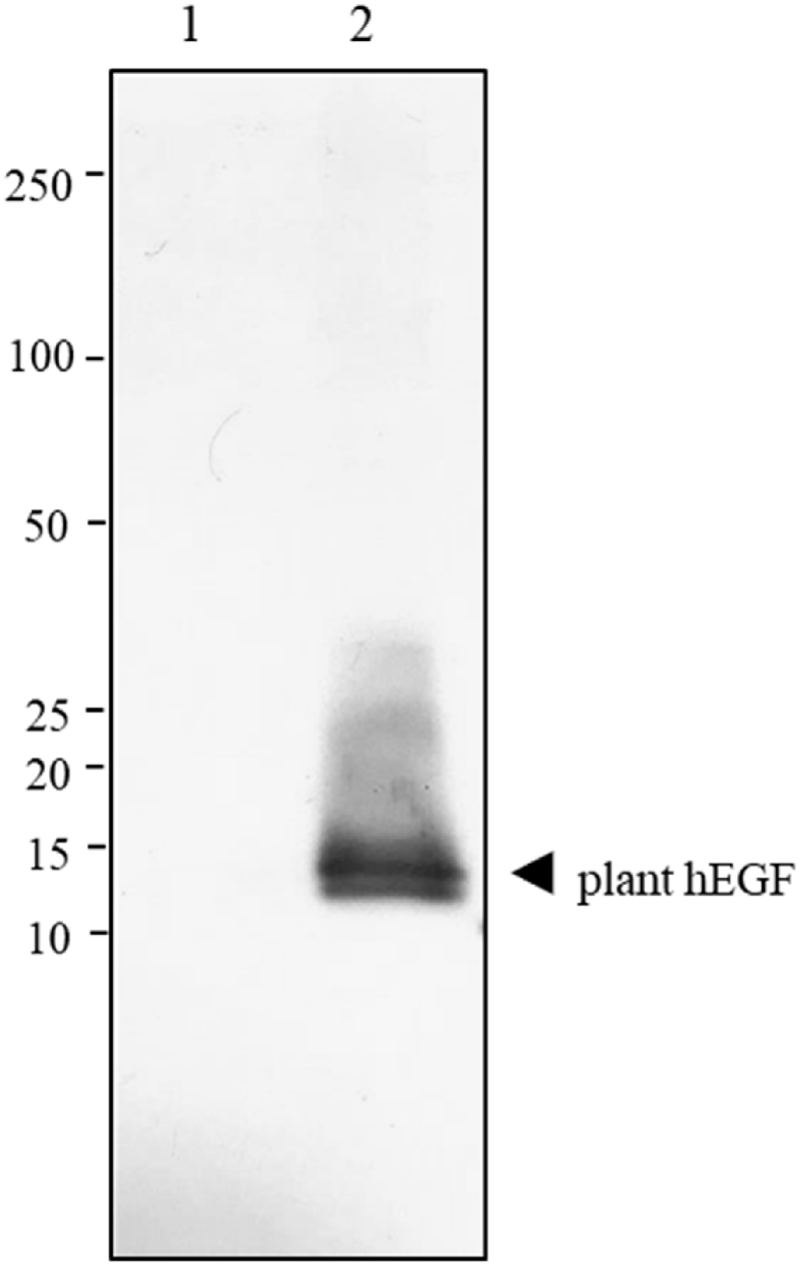
Western blot analysis of purified plant-produced hEGF under reducing condition. The blot was probed with mouse anti-human EGF antibody. Lane 1, non-infiltrated wild-type *N. benthamiana* crude extract Lane 2, purified plant-produced hEGF.

A single-step Ni-NTA affinity chromatography was performed to purify hEGF from plant crude extract. The size and identity of the plant-produced hEGF was confirmed by SDS-PAGE and Western blot analysis probed with mouse anti-human EGF antibody (R&D system, USA) under reducing condition (Fig. 4). Furthermore, we have also attempted to extend the preliminary investigation on optimizing the purification condition in order to improve protein quality and yield.

### 3.4 Improving the purification efficiency of Ni-NTA affinity chromatography

Recombinant protein production from plants relies on protein extraction which is the first and important step to collect the protein of interest in form of crude extract followed by purification to recover the target protein. The hEGF gene construct was fused with His tag to facilitate the purification through the selective interaction with the Ni-NTA affinity chromatography.

#### 3.4.1 Effect of extraction volume

**Fig. 5:**
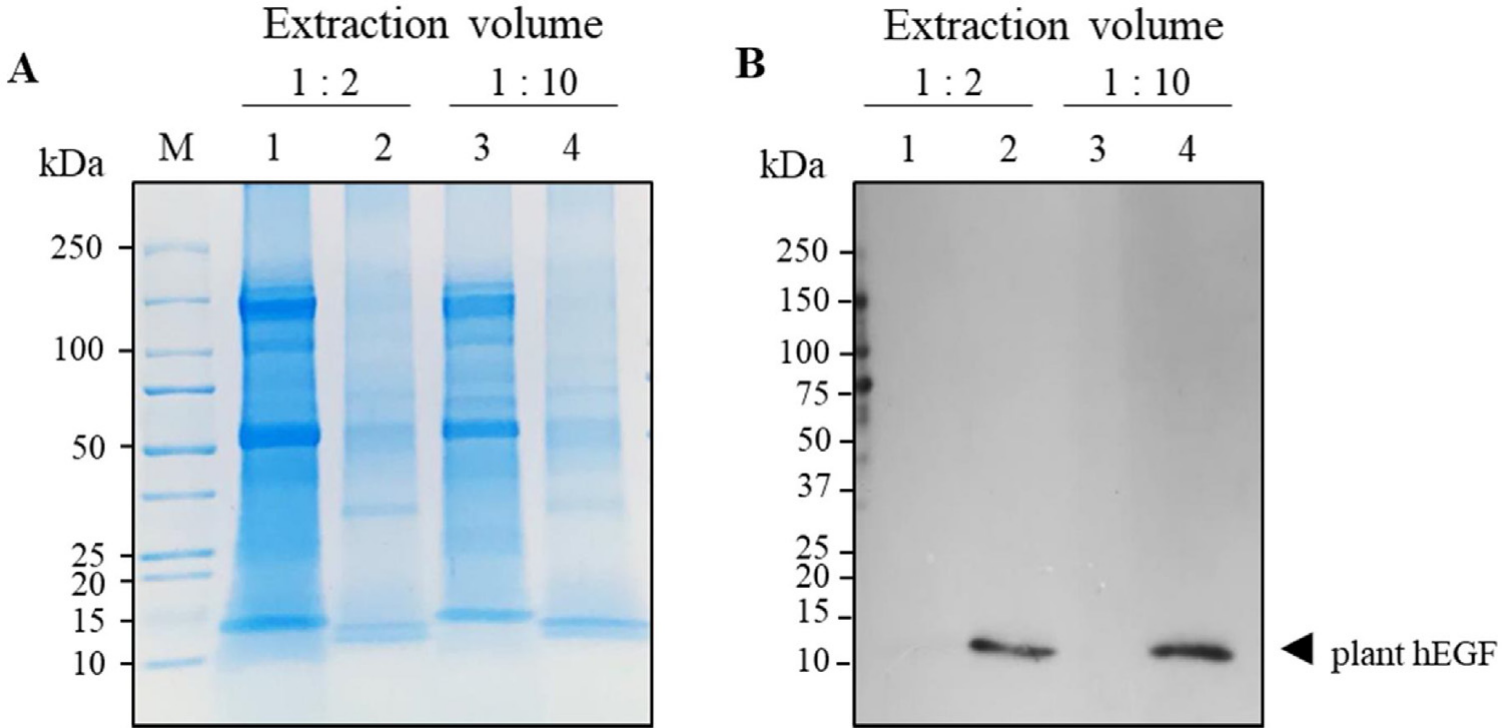
Effect of extraction buffer concentration on protein purification (A) SDS-PAGE analysis of plant crude extract extracted from different concentration of extraction buffer. (B) Western blot of plant-produced hEGF crude extract under reducing condition. The plant-produced hEGF sample was extracted from the same harvested leaves by using different extraction buffer concentration (1:2 and 1:10). kDa: kilodalton; M: protein marker, Lane 1, 3: total soluble protein from plant crude extracts extracted with 1:2 and 1:10 extraction buffer concentration, respectively; Lane 2, 4: purified hEGF protein eluted from Ni-NTA affinity chromatography. Arrowhead represents plant-produced hEGF.

A preliminary attempt to remove host cell contaminants during purification was evaluated. We examined the impact of high volume of crude extract in enhancing the purification efficiency during Ni-NTA affinity chromatography. As shown in Fig. 5A, coomassie blue-stained gels indicated that the protein profiles of eluted protein collected from the higher dilution volume of plant crude extract after purification (1:10) was more than the eluted protein collected from the lower dilution volume of plant crude extract (1:2). Furthermore, the band intensity of the hEGF recovered (Fig. 5B) from high volume (1:10) was found to be slightly higher compared to the other one, which indicates that the high extraction volume allows the Ni-NTA affinity resin to recover his-tagged protein from plant crude extract effectively.

#### 3.4.2 Effect of ammonium sulfate precipitation

**Fig. 6:**
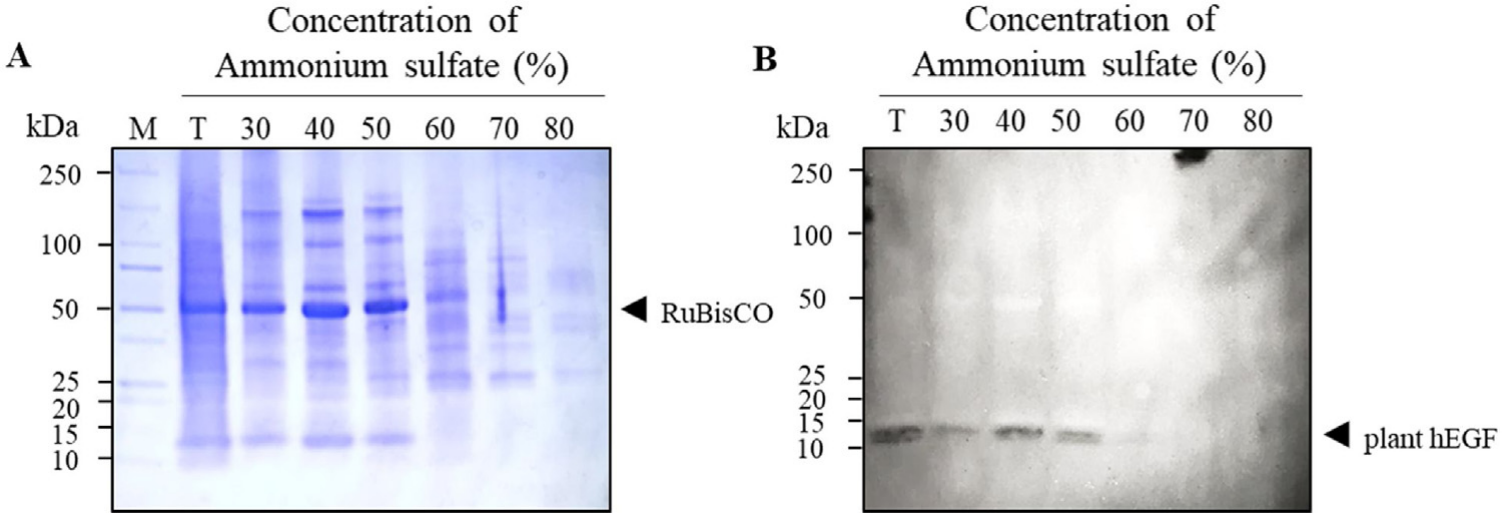
Effect of ammonium sulfate concentration on protein precipitation and purification: (A) SDS-PAGE analysis of precipitated proteins collected from 30-80 % saturated ammonium sulfate concentration in plant crude extract (B) Western blot of plant-produced hEGF crude extract under reducing condition. The blot was probed with HRP-conjugated anti-His antibody. kDa: kilodalton; T: total soluble protein from plant crude extract; the lane number represents the ammonium sulfate concentration used for precipitating the proteins. Arrowhead indicates RuBisCO (Fig. 6A) and plant-produced hEGF (Fig. 6B).

The effect of ammonium sulfate concentration on precipitation of TSP after protein extraction was evaluated. The precipitated TSP was resolved in SDS gel and visualized by staining with coomassie brilliant blue. The results showed that 30, 40, and 50 % of ammonium sulfate effectively precipitates most of the RuBisCO protein as pellets (Fig. 6A). However, the higher concentration of the plant-produced hEGF protein was visualized in the gel at the ammonium sulfate saturation level of 30–50 %, indicated that the recombinant hEGF was co-precipitated along with RuBisCO, whereas low or no proteins were detected at 60 % and 70–80 % ammonium sulfate concentration respectively (Fig. 6B). This observation suggested that ammonium sulfate precipitation could partially remove host cell contaminants from the plant-produced hEGF; however further process optimization is highly essential to improve the protein quality.

#### 3.4.3 Effect of extraction buffer pH

**Fig. 7:**
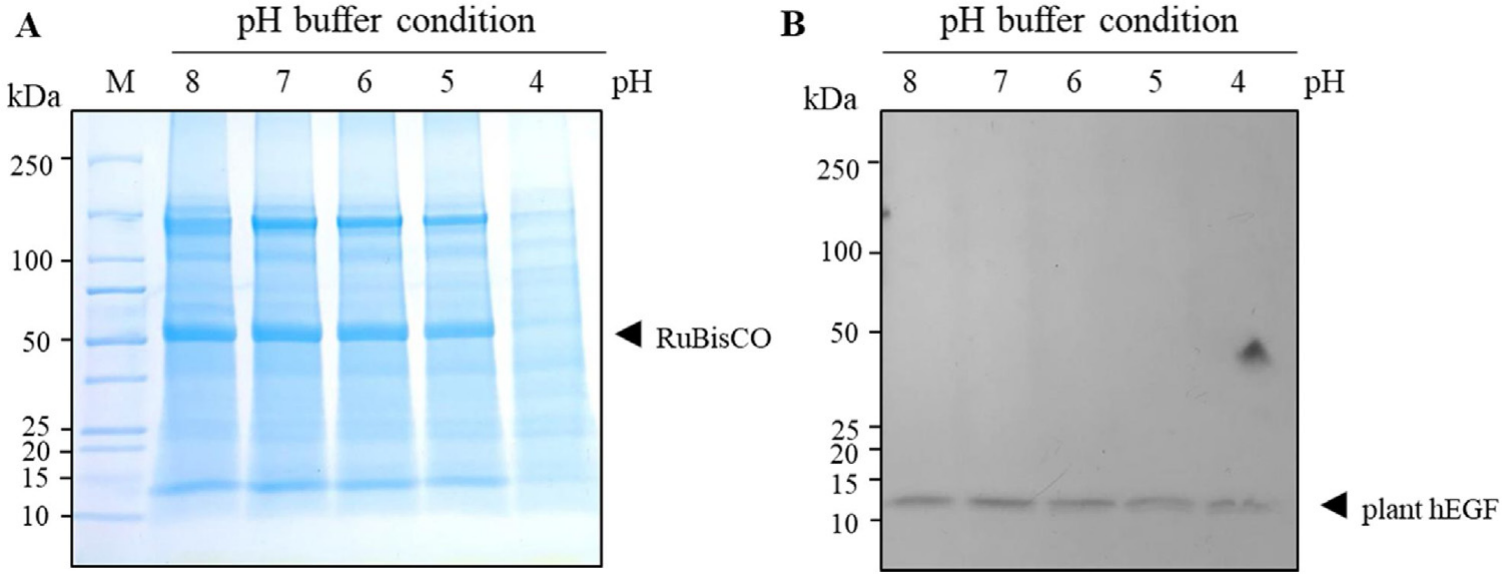
Effect of extraction buffer pH on protein purification: Protein purity improvement of plant-produced hEGF extracted with different pH of extraction buffer. One gram of fresh weight was extracted with different pH extraction buffer (pH 4, 5, 6, 7, and 8). (A) SDS-PAGE analysis of plant crude extract extracted from different extraction buffer pH (B) Western blot of plant-produced hEGF crude extract under reducing condition. The blot was probed with HRP-conjugated anti-His antibody. kDa: kilodalton; the lane number represents the pH of extraction buffer used for removing host protein contaminants from plant crude extract. Arrowhead indicates RuBisCO (Fig. 7A) and plant-produced hEGF (Fig. 7B).

The harvested plant leaves were homogenized in different extraction buffers with varying pH ranges from pH 4 to 8. As shown in Fig. 7A, coomassie blue-stained gel showed a prominent ~ 50 kDa band of RuBisCO protein was largely removed from the TSP extracted by using extraction buffer pH 4. Further, the band intensity of hEGF from pH 4 buffer was slightly reduced compared to other pH buffer conditions (Fig. 7B). These results showed that low pH extraction buffer could largely remove the host cell contaminants, especially the abundant RuBisCO proteins from the crude extract which could improve the performance efficiency of subsequent purification step.

### 3.5 Cytotoxicity and cell proliferation assay

**Fig. 8:**
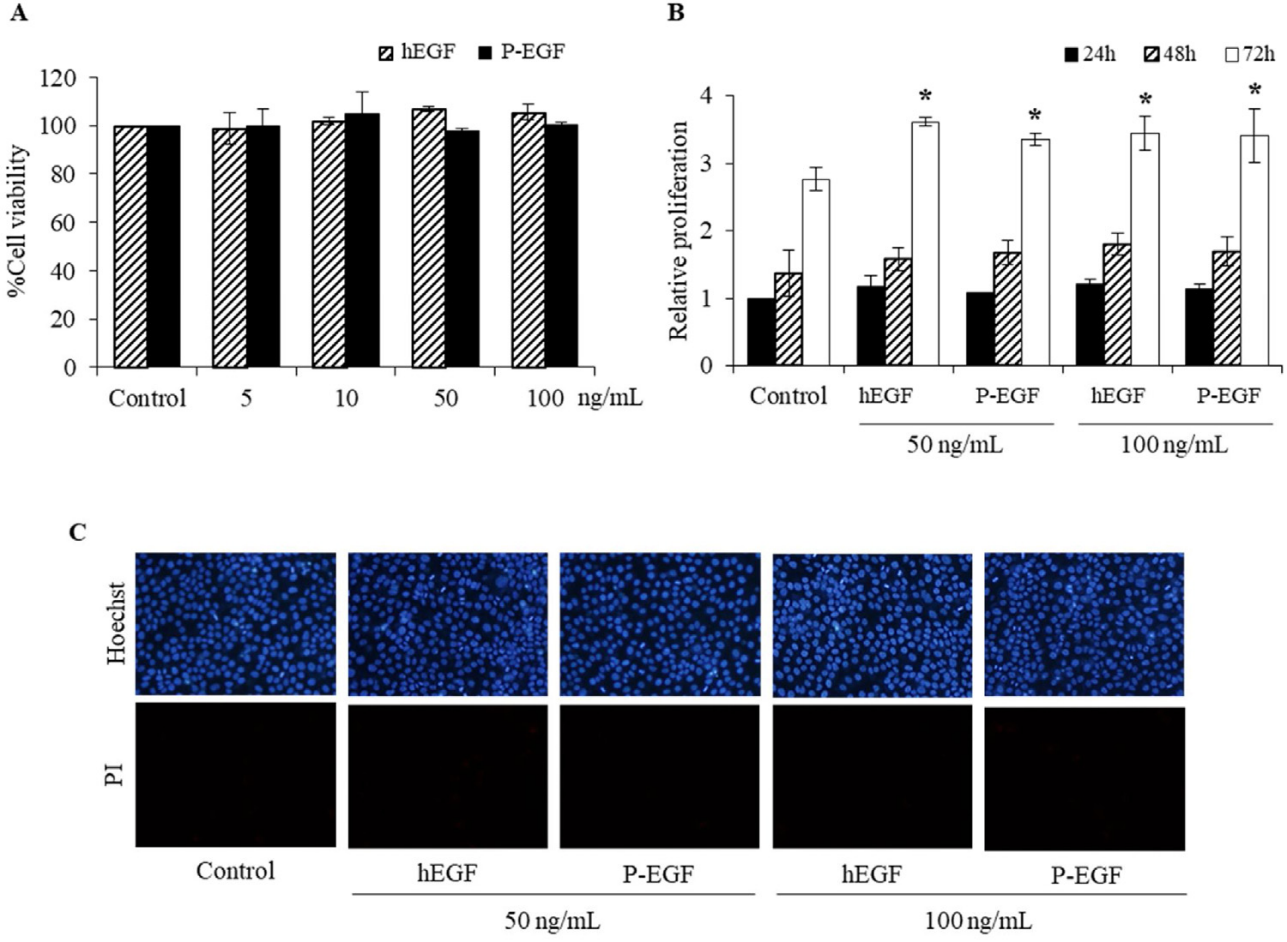
Cell proliferation and cytotoxic effect of plant-produced hEGF in HaCaT cells analyzed by MTT assay (A) After 24 h of treatment with plant-produced EGF (P-EGF) and commercial hEGF (hEGF-control) at the concentration from 5, 10, 50 and 100 ng/mL. Values are shown as mean ± SD of the percentage of HaCaT cell viability. (B) Concentration of hEGF at 50, 100 ng/mL did not affect HaCaT cell proliferation. (C) Visualization of HaCaT co-stained with Hoechst 33,342 and PI dye showed that no apoptotic or necrotic cell death was observed after treatment with hEGF.

Cytotoxic effect of hEGF expressed in *N. benthamiana* was assessed in human keratinocyte cell (HaCaT) by MTT assay. Cytotoxicity assay was performed by treatment with purified plant-produced hEGF (P-EGF) and commercial hEGF (hEGF-Control).The different concentrations of purified plant-produced hEGF from 5−100 ng/mL were tested. As shown in Fig. 8A, the plant-produced hEGF did not exhibit any cytotoxic effects similar to commercial hEGF in all the tested concentrations. Further, the proliferative effect of plant-produced hEGF was also determined on HaCaT cell. Fig. 8B illustrates that hEGF at 50, 100 ng/mL concentrations induce HaCaT cell proliferation. Hence, these concentrations were selected for further experiments.

Human EGF-treated HaCaT cells were further analyzed with co-staining of Hoechst 33342 and PI dye to assess cell death. As shown in Fig. 8C, no apoptotic or necrotic cells were detected in the hEGF-treated cells irrespective of the concentration tested.

### 3.6 Cell migration effect of recombinant hEGF

**Fig. 9:**
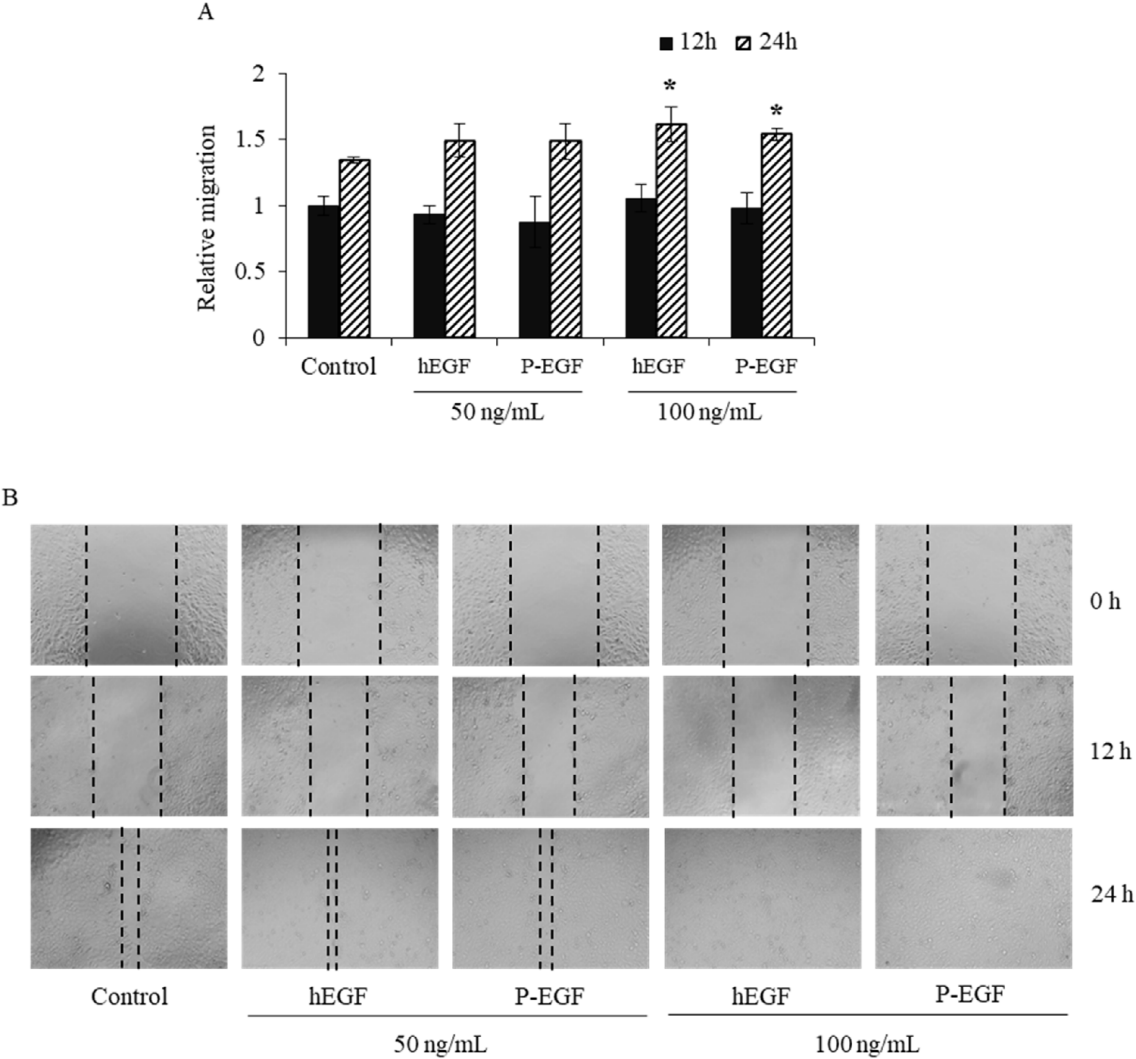
Migration effect of plant-produced hEGF on HaCaT cells (A) Relative migration after 12 and 24 h treatment with 50, 100 ng/mL of plant-produced EGF (P-EGF) and commercial hEGF (hEGF-control). The data was presented as mean ± SD; * indicates a significant difference of relative migration level between treated cells and untreated cells at *p ≤ 0.05*. (B) The closure of wound area was observed at 12 and 24 h after treatment with 50, 100 ng/mL of plant-produced and commercial hEGF.

The migratory activity of HaCaT cells treated with plant-produced hEGF was evaluated by *in vitro* scratch assay (Fig. 9A, B). HaCaT cells were seeded onto a 96-well plate, and then cultured until the formation of the confluent monolayer. The cell monolayer was scratched to generate the wound area and treated with 50, 100 ng/mL of plant-produced hEGF. The closure of wound area was monitored for 0, 12, and 24 h. The relative migration of wound area at 12 and 24 h was measured. The results showed that HaCaT cell migration was significantly induced after treatment with 100 ng/mL of plant-produced hEGF and commercial hEGF when compared with non-treated cells as negative control.

## 4 Discussion

Plant molecular farming (PMF) has been evaluated extensively as a potential expression platform for the production of biopharmaceutical proteins including therapeutic proteins, growth factors, enzymes, antibodies, and vaccines [15]. *N. benthamiana* is employed extensively for PMF applications and has been considered as a preferred host by many plant-based pharma companies including Mapp Biopharmaceutical, Inc., Icon Genetics GmbH, Kentucky BioProcessing, and Medicago, *etc.*

The generation of recombinant growth factors becomes an interest of the biopharmaceutical industry due to its potential application in regenerative medicine [26]. A range of growth factors including acid fibroblast growth factor (aFGF) [27,28], keratinocyte growth factor 1 (KGF1) [29], human epidermal growth factor (hEGF) [21,30] where produced in plants. hEGF was expressed in different plant hosts such as tomato [31], soybean [32], and also in *Nicotiana* species [21,22]. Zhi et al. (2007) reported the EGF yield of up to 3.48 ± 1.01 ng/g fresh weight in transgenic tomato expressing the codon-optimized EGF; whereas the yield of up to 6.7 ± 3.1–129.0 ± 36.7 μg EGF per gram dry seed was reported in transgenic soybean [32]. However, developing a stable transgenic line requires a long period of time (approximately 3–6 months) to generate homozygous plants [33,34]. It is important that the transient expression of hEGF in *N. benthamiana* could rapidly produce large quantities of protein in less than one week after plant transformation.

In this study, we aim to enhance the hEGF protein accumulation by optimizing parameters such as effective gene construct design and *Agrobacterium* cell density. In addition, a preliminary study was attempted to increase the protein purification efficiency. The hEGF coding sequence was codon-optimized and cloned into a geminiviral replicon vector [35]. The vector was modified from the bean yellow dwarf virus which was shown to provide rapid and high mRNA levels for the high expression of protein of interest [35,36].

With regard to gene construct design, the hEGF construct, flanked with N-terminal signal peptide and C-terminal His tag, SEKDEL motif (SP-EGF-H-KD), exhibited the highest expression level compared to other tested constructs. The results showed that the two C-terminal His tagged constructs (SP-EGF-H-KD and EGF-H) were detected by the anti-His antibody. None of the hEGF constructs flanked with N-terminal His tagged protein were detected under reducing condition in Western blot which might be due to partial protein digestion or protein folding [37]. The highest level of hEGF expression up to 15.695 μg/g leaf fresh weight or 0.499 % TSP was obtained with the optimized construct. The increased expression might be due to the codon optimization and ER retention motif, similar to that reported by Bai et al. 2007 [22]. Many reports have illustrated the positive impact of targeting proteins to the ER compartment in terms of elevated recombinant protein accumulation and protein quality [[38], [39], [40]].

Transformation conditions are key elements affecting the transformation efficiency which in turn affect the protein expression. *Agrobacterium* concentration or cell density is one of such factors that affect the efficiency of plant transformation; low concentration may result in insufficient *Agrobacterium* infection, whereas high concentration can cause the plant cell damage or trigger hypersensitive response leading to plant tissue necrosis [41,42]. We found that *Agrobacterium* cell density at the OD_600_ of 0.2 was found to be optimal for plant transformation. However, *Agrobacterium* concentration needs to be optimized for different proteins and the selection of optimal cell density should balance the maximum transformation efficiency and tissue necrosis that occurs due to many contributing factors such as nature of the protein, sub-cellular localization, and expression levels *etc* [36].

The recovery of the target protein from total soluble protein in the plant crude extracts determines the efficiency of the production system. However, after purification, depending on the recombinant protein expression level and the nature of host cell contaminants, sometimes non-specific proteins can also be found along with the purified proteins [43]. In case of *E.coli* based production platform, protein purity of more than 80 % has been reported after one chromatographic step [43].The native host proteins in *Nicotiana*-based platform are commonly released during cell disruption for protein extraction [24], which might probably obscure the binding interaction of recombinant protein to the affinity matrices in chromatographic step. Thereby, increasing the contaminants in elution fraction [44]. Affinity chromatography is one of the most convenient methods used for recombinant protein purification. In this study, the hEGF vector constructs were fused with His tag in order to facilitate the recombinant protein purification from plant crude extracts by Ni-NTA affinity chromatography. With a His tag and a SEKDEL ER retention signal, a band at approximately 12 kDa was observed in western blot which is slightly higher than the expected molecular size of 8 kDa. However, the identity of the plant-produced protein was established by anti-human EGF antibody which is directed specifically against EGF.

Sometimes, the his-tagged target protein might be co-purified alongwith abundant host protein carrying at least two adjacent histidine residues which commonly found in some of the host cellular proteins [45,46]. These contaminants could interfere with the interaction of Ni-NTA resin with the target protein resulting in low yield/purity of the target protein. Hence a preliminary investigation on improving the purification efficiency of recombinant protein from plant crude extract was attempted. Higher dilution of extraction volume (1:10) was shown to have a positive effect on improving the protein purity and recovery. Although a substantial amount of host contaminants are still co-eluted into the purified protein, we continued investigating other strategies to improve the purification efficiency. Ammonium sulfate precipitation is commonly used as an initial purification step to remove proteins that easily aggregated under high salt conditions [47]. This method has been utilized to remove plant host proteins such as the photosynthetic protein, RuBisCO, other aggregated proteins, and cell debris [24]. Saturated ammonium sulfate at 40 % concentration was effective in precipitating most RuBisCO and other plant proteins; but hEGF was also precipitated alongside with other proteins in ammonium sulfate concentration step which implies the need for this process optimization further. Similar to ammonium sulfate precipitation, the effect of low pH extraction buffer was also investigated to remove host protein in the crude extract. Although the hEGF band intensity reduced in low pH extraction buffer (pH 4 and pH 5) probably owing to less extractable hEGF from plant tissues, reduced solubility, or protein degradation, the prominent RuBisCO protein was largely removed by low pH extraction buffer (pH 4) from crude extract. However, rigorous experimentations are required to support these preliminary results and also further purification optimization, scale-up experiments are needed to improve the quantity and quality of recombinant proteins to ascertain an industrial manufacturing process that could be helpful to realize the benefits of plant expression system.

Human EGF has been assessed for its biological activity on cell proliferation in epidermal cells [1], fibroblast [48], and keratinocytes [49]. Here, we assessed the biological activity of plant-produced hEGF on human keratinocytes HaCaT cells. Our data showed that no cytotoxicity was observed when treating the cells with the recombinant plant-produced hEGF similar to commercial hEGF up to the highest tested concentration of 100 ng/mL. Moreover, plant-produced hEGF was shown to enhance the HaCaT cell proliferation and migration in a dose-dependent manner. A significant increase in relative cell migration was observed at the hEGF concentration of 100 ng/mL.

In summary, we have evaluated the effective vector design and *Agrobacterium* cell density to improve recombinant hEGF production in *N. benthamiana*. The conditions were optimized and the recombinant hEGF was expressed in *N. benthamiana* leaves *via.,* transient expression which results in high protein accumulation within 4 dpi. In addition, we also demonstrated the cell proliferation and cell migration effect of the plant-produced hEGF *in vitro*. Altogether, these results indicated the potential of plant expression system for the production of recombinant EGF which can be exploited in tissue engineering and cosmeceuticals.

## CRediT authorship contribution statement

**Oranicha Hanittinan:** Methodology, Writing - original draft, Writing - review & editing. **Yamin Oo:** Methodology, Writing - original draft, Writing - review & editing. **Chatchai Chaotham:** Methodology, Writing - original draft, Writing - review & editing. **Kaewta Rattanapisit:** Methodology. **Balamurugan Shanmugaraj:** Methodology, Writing - original draft, Writing - review & editing, Supervision. **Waranyoo Phoolcharoen:** Methodology, Writing - original draft, Writing - review & editing, Supervision, Funding acquisition.

## Declaration of Competing Interest

The authors declare no conflicts of interest.

## References

[1] Cohen S., Elliott G.A. (1963). The stimulation of epidermal keratinization by a protein isolated from the submaxillary gland of the mouse. J. Invest. Dermatol..

[2] Cohen S. (1962). Isolation of a mouse submaxillary gland protein accelerating incisor eruption and eyelid opening in the new-born animal. J. Biol. Chem..

[3] Zeng F., Harris R.C. (2014). Epidermal growth factor, from gene organization to bedside. Seminars in Cell & Developmental Biology.

[4] Aldag C., Teixeira D.N., Leventhal P.S. (2016). Skin rejuvenation using cosmetic products containing growth factors, cytokines, and matrikines: a review of the literature. Clin. Cosmet. Investig. Dermatol..

[5] Ziegler T.R., Pierce G.F., Herndon D.N. (2012). Growth Factors and Wound Healing: Basic Science and Potential Clinical Applications.

[6] Wu H.G., Song S.Y., Kim Y.S., Oh Y.T., Lee C.G., Keum K.C., Ahn Y.C., Lee Sw. (2009). Therapeutic effect of recombinant human epidermal growth factor (RhEGF) on mucositis in patients undergoing radiotherapy, with or without chemotherapy, for head and neck cancer: a double‐blind placebo‐controlled prospective phase 2 multi‐institutional clinical trial. Cancer.

[7] Sivakesava S., Xu Z., Chen Y., Hackett J., Huang R., Lam E., Lam T., Siu K., Wong R., Wong W. (1999). Production of excreted human epidermal growth factor (hEGF) by an efficient recombinant Escherichia coli system. Process Biochem..

[8] Yamagata H., Nakahama K., Suzuki Y., Kakinuma A., Tsukagoshi N., Udaka S. (1989). Use of Bacillus brevis for efficient synthesis and secretion of human epidermal growth factor. PNAS.

[9] George-Nascimento C., Gyenes A., Halloran S.M., Merryweather J., Valenzuela P., Steimer K.S., Masiarz F.R., Randolph A. (1988). Characterization of recombinant human epidermal growth factor produced in yeast. Biochemistry.

[10] Mohammadian J., Mansoori-Derakhshan S., Mohammadian M., Shekari-Khaniani M. (2013). Construction of yeast recombinant expression vector containing human epidermal growth factor (hEGF). Adv. Pharm. Bull..

[11] Negahdari B., Shahosseini Z., Baniasadi V. (2016). Production of human epidermal growth factor using adenoviral based system. Res. Pharm. Sci..

[12] Berkmen M. (2012). Production of disulfide-bonded proteins in Escherichia coli. Protein Expres. Purif..

[13] Fischer R., Buyel J.F. (2020). Molecular farming–the slope of enlightenment. Biotechnol. Adv..

[14] Schillberg S., Raven N., Spiegel H., Rasche S., Buntru M. (2019). Critical analysis of the commercial potential of plants for the production of recombinant proteins. Front. Plant Sci..

[15] Obembe O.O., Popoola J.O., Leelavathi S., Reddy S.V. (2011). Advances in plant molecular farming. Biotechnol. Adv..

[16] Shanmugaraj B., Ramalingam S. (2014). Plant expression platform for the production of recombinant pharmaceutical proteins. Austin. J. Biotechnol. Bioeng..

[17] Zhang B., Shanmugaraj B., Daniell H. (2017). Expression and functional evaluation of biopharmaceuticals made in plant chloroplasts. Curr. Opin. Chem. Biol..

[18] Conley A.J., Zhu H., Le L.C., Jevnikar A.M., Lee B.H., Brandle J.E., Menassa R. (2011). Recombinant protein production in a variety of *Nicotiana* hosts: a comparative analysis. Plant Biotechnol. J..

[19] Shanmugaraj B., Bulaon C.J.I., Phoolcharoen W. (2020). Plant molecular farming: a viable platform for recombinant biopharmaceutical production. Plants.

[20] Alkanaimsh S., Corbin J.M., Kailemia M.J., Karuppanan K., Rodriguez R.L., Lebrilla C.B., McDonald K.A., Nandi S. (2019). Purification and site-specific N-glycosylation analysis of human recombinant butyrylcholinesterase from *Nicotiana benthamiana*. Biochem. Eng. J..

[21] Wirth S., Calamante G., Mentaberry A., Bussmann L., Lattanzi M., Barañao L., Bravo-Almonacid F. (2004). Expression of active human epidermal growth factor (hEGF) in tobacco plants by integrative and non-integrative systems. Mol. Breed..

[22] Bai J.-Y., Zeng L., Hu Y.-L., Li Y.-F., Lin Z.-P., Shang S.-C., Shi Y.-S. (2007). Expression and characteristic of synthetic human epidermal growth factor (hEGF) in transgenic tobacco plants. Biotechnol. Lett..

[23] Thomas D.R., Walmsley A.M. (2014). Improved expression of recombinant plant-made hEGF. Plant Cell Rep..

[24] Wilken L.R., Nikolov Z.L. (2012). Recovery and purification of plant-made recombinant proteins. Biotechnol. Adv..

[25] Atale N., Gupta S., Yadav U., Rani V. (2014). Cell‐death assessment by fluorescent and nonfluorescent cytosolic and nuclear staining techniques. J. Microsc..

[26] Mitchell A.C., Briquez P.S., Hubbell J.A., Cochran J.R. (2016). Engineering growth factors for regenerative medicine applications. Acta Biomater..

[27] Liu J., Ma P., Sun Y., Yang M., Yang L., Li Y., Wu Y., Zhu X., Wang X. (2007). Expression of human acidic fibroblast growth factor in *Nicotiana benthamiana* with a potato‐virus‐X‐based binary vector. Biotechnol. Appl. Biochem..

[28] Ha J.-H., Kim H.-N., Moon K.-B., Jeon J.-H., Jung D.-H., Kim S.-J., Mason H.S., Shin S.-Y., Kim H.-S., Park K.-M. (2017). Recombinant human acidic fibroblast growth factor (aFGF) expressed in *Nicotiana benthamiana* potentially inhibits skin photoaging. Planta Med..

[29] Feng Z.-G., Pang S.-F., Guo D.-J., Yang Y.-T., Liu B., Wang J.-W., Zheng K.-Q., Lin Y. (2014). Recombinant keratinocyte growth factor 1 in tobacco potentially promotes wound healing in diabetic rats. Biomed Res. Int..

[30] Zhao H., Tan Z., Wen X., Wang Y. (2017). An improved syringe agroinfiltration protocol to enhance transformation efficiency by combinative use of 5-azacytidine, ascorbate acid and tween-20. Plants.

[31] Zhi Q., Wang S., Chai M., Zhang F., Li Q., Li S., Sun M. (2007). Transgenic mini-tomato and protection against alcohol-induced gastric injury. J. Genet. Genomics.

[32] He Y., Schmidt M.A., Erwin C., Guo J., Sun R., Pendarvis K., Warner B.W., Herman E.M. (2016). Transgenic soybean production of bioactive human epidermal growth factor (EGF). PLoS One.

[33] Yao J., Weng Y., Dickey A., Wang K. (2015). Plants as factories for human pharmaceuticals: applications and challenges. Int. J. Mol. Sci..

[34] Xu J., Towler M., Weathers P.J. (2018). Platforms for plant-based protein production. Bioproces. Plant in vitro Syst..

[35] Chen Q., He J., Phoolcharoen W., Mason H.S. (2011). Geminiviral vectors based on bean yellow dwarf virus for production of vaccine antigens and monoclonal antibodies in plants. Hum. Vaccin..

[36] Mason H.S., Diamos A.G. (2018). Modifying the replication of Geminiviral vectors reduces cell death and enhances expression of biopharmaceutical proteins in *Nicotiana benthamiana* leaves. Front. Plant Sci..

[37] Debeljak N., Feldman L., Davis K.L., Komel R., Sytkowski A.J. (2006). Variability in the immunodetection of His-tagged recombinant proteins. Anal. Biochem..

[38] Heidari H.R., Bandehpour M., Vahidi H., Barar J., Kazemi B., Naderi-Manesh H. (2014). Improvement in the stability and functionality of *Nicotiana tabacum* produced recombinant TRAIL through employment of endoplasmic reticulum expression and ascorbate buffer mediated extraction strategies. BioImpacts.

[39] Petruccelli S., Otegui M.S., Lareu F., Tran Dinh O., Fitchette A.C., Circosta A., Rumbo M., Bardor M., Carcamo R., Gomord V. (2006). A KDEL‐tagged monoclonal antibody is efficiently retained in the endoplasmic reticulum in leaves, but is both partially secreted and sorted to protein storage vacuoles in seeds. Plant Biotechnol. J..

[40] Benchabane M., Goulet C., Rivard D., Faye L., Gomord V., Michaud D. (2008). Preventing unintended proteolysis in plant protein biofactories. Plant Biotechnol. J..

[41] Yan R., Wang Z., Ren Y., Li H., Liu N., Sun H. (2019). Establishment of efficient genetic transformation systems and application of CRISPR/Cas9 genome editing technology in Lilium pumilum DC. Fisch. and Lilium longiflorum White Heaven. Int. J. Mol. Sci..

[42] Leuzinger K., Dent M., Hurtado J., Stahnke J., Lai H., Zhou X., Chen Q. (2013). Efficient agroinfiltration of plants for high-level transient expression of recombinant proteins. J. Vis. Exp..

[43] Kimple M.E., Brill A.L., Pasker R.L. (2013). Overview of affinity tags for protein purification. Curr. Protoc. Protein Sci..

[44] Buyel J., Twyman R., Fischer R. (2015). Extraction and downstream processing of plant-derived recombinant proteins. Biotechnol. Adv..

[45] Mbewana S., Meyers A.E., Weber B., Mareledwane V., Ferreira M.L., Majiwa P.A., Rybicki E.P. (2018). Expression of Rift Valley fever virus N-protein in *Nicotiana benthamiana* for use as a diagnostic antigen. BMC Biotechnol..

[46] Bornhorst J.A., Falke J.J. (2000). Purification of proteins using polyhistidine affinity tags. Methods in Enzymology.

[47] Duong-Ly K.C., Gabelli S.B., Lorsch J. (2014). Chapter seven - salting out of proteins using ammonium sulfate precipitation. Methods in Enzymology.

[48] Lembach K.J. (1976). Induction of human fibroblast proliferation by epidermal growth factor (EGF): enhancement by an EGF-binding arginine esterase and by ascorbate. PNAS.

[49] Barrandon Y., Green H. (1987). Cell migration is essential for sustained growth of keratinocyte colonies: the roles of transforming growth factor-α and epidermal growth factor. Cell.

